# Failure of the Law to Grant Access to Legal Abortion in Chile

**DOI:** 10.1089/heq.2023.0050

**Published:** 2024-03-20

**Authors:** Daniel F.M. Suárez-Baquero, Ilana G. Dzuba, Mariana Romero, C. Finley Baba, M. Antonia Biggs

**Affiliations:** ^1^ Department of Child, Family, and Population Health, School of Nursing, University of Washington, Seattle, Washington, USA.; ^2^ Postdoctoral Fellow ACTIONS Program, Department of Family Health Care Nursing, School of Nursing, University of California San Francisco, San Francisco, California, USA.; ^3^ Gynuity Health Projects, New York, New York, USA.; ^4^ National Abortion Federation, Washington, District of Columbia, USA.; ^5^ Advancing New Standards in Reproductive Health, University of California San Francisco, Oakland, California, USA.

**Keywords:** Chile, abortion, abortion access, qualitative research

## Abstract

**Introduction:**

In 2017, Chile decriminalized abortion on three grounds: (i) if the pregnant person's life is at risk, (ii) fetal nonviability, and (iii) rape or incest. This multicase study explores the experiences of pregnant people legally entitled to but denied access to legal abortion in Chile.

**Methods:**

Through a snowball sampling approach, we recruited adult Chilean residents who sought, were eligible for, and were denied a legal abortion after September 2017. We conducted semistructured interviews with participants to explore their experiences in seeking and being denied legal abortions. We recorded and transcribed the interviews, then coded and analyzed the transcriptions to identify common themes.

**Results:**

We identified four women who met the eligibility criteria. The interviews revealed five common themes in their experiences: (i) disparate levels of social support in accessing abortion, (ii) abundant access barriers, (iii) forced pregnancy, (iv) abortion stigma, and (v) a failure of the law to provide access to abortion.

**Discussion and Health Equity Implications:**

Although the 2017 law expanded legal access to abortion in Chile, significant barriers remain. Compounded with social stigma, and the socioeconomic disparities in abortion access, pregnant people continue to face insurmountable obstacles in obtaining legal abortions, even when their lives are at risk and the pregnancy is not viable. The state must prioritize equity of access to legal abortions. Future studies should continue to explore the challenges people face accessing legal abortion care to inform strategies to ensure people are able to obtain the quality care that they are legally entitled to.

## Introduction

From 1989 to 2017, Chile was one of the few countries that banned abortion without legal exception.^1^ On August 21, 2017, Chile made an important legal shift and decriminalized abortion on three grounds: (i) risk to the pregnant person's life, (ii) fetal nonviability, and (iii) rape or incest; estimated to provide a legal option for only 3% (*n*=2550) of the abortions annually.^2^ However, Chile's Ministry of Health (MOH) data suggest that the actual number of abortions granted on legal grounds was only a small fraction, about half, of the original estimates.^3–5^ The lower rate of legal abortions was likely due, in part, to the medically unnecessary legal requirement that abortions be performed by licensed physicians in facilities with high-risk obstetric units, as well as the high number of institutions and physicians who claimed conscientious objection. Initial data suggested that most obstetric gynecologist (OB/GYN) specialists claimed conscientious objection, with nearly half of those practicing in public hospitals refusing to provide care to people seeking an abortion due to rape.^6^

While abortion in Chile is only legally permitted in limited circumstances, it is well known that thousands of people have abortions outside of the formal health care sector, oftentimes with the support of accompaniment groups. According to the Guttmacher Institute, between 2015 and 2019, there were ∼170,000 pregnancies that ended in abortion.^7^ Many of these abortions involved the use of medications up to 24 weeks gestation.^8^ According to a retrospective analysis of anonymized case records from accompaniment groups based in Argentina, Chile, and Ecuador, from 2016 to 2018, 316 individuals had safe and effective medication abortions between the 13th and 24th weeks of pregnancy, using a combination of mifepristone-misoprostol regimen.

These findings demonstrated the safety and effectiveness of self-managed medication abortions supported by an accompaniment model for abortions both early and later in pregnancy, especially in regions with legal constraints.^8^ Similarly, another recent study found that self-managed abortion was safe, effective, and acceptable among people who had abortions outside of the formal health care sector in Chile and received less judgmental, more supportive and informative services than they had received within the formal health care setting.^9^

An exploration into women's lived experiences trying to access abortion in Chile offers a compelling vantage point from which to assess the implementation of abortion laws globally. The intricate tapestry of abortion legislation in Latin America encompasses diverse approaches, ranging from more progressive policies in countries like Colombia, Cuba, and Mexico, that decriminalize all abortions up to 24 weeks gestation and beyond 24 weeks for abortions due to rape and maternal or fetal health indications to Uruguay and Argentina which allow abortion under specific circumstances, to complete bans on abortion in El Salvador and Nicaragua, where people involved in suspected abortions can be punished by up to 8 years in prison. This regional variation underscores the intricate interplay of sociopolitical factors that shape abortion laws and ensure or deny abortion access.

Chile's move to expand access to abortion contrasts with recent developments in the United States and Poland that have moved to restrict access to abortion. In the United States, the decision that overturned the landmark Roe v. Wade case removed federal protections on abortion, unraveling decades of abortion rights progress and highlighting the fragility of reproductive rights even in established democracies.

The central aim of this study is to detail individuals' experiences accessing legal abortion care in Chile soon after decriminalization. Through this multiple-case study, we document the lived experiences of four people who identified as women yet found themselves unjustly denied the services they were unequivocally entitled to. By delving deep into these personal narratives, our research aims to better understand women's experiences seeking and being denied care, including the barriers faced, pregnancy outcomes, and their experiences seeking care outside the legal framework. By sharing their stories we reveal challenges in the implementation of legal reform and provide insight in how to improve access.

## Methods

This exploratory study aimed to interview people who were eligible for (under the three legal grounds) but unable to access legal abortion services in Chile after legal reform (September 14, 2017). To be eligible, participants had to speak Spanish, be 18 years or older, live in Chile, and to have been denied legal access to abortion.

We recruited participants from May 2019 to April 2020 using a snowball sampling approach, including referrals from health care providers, MOH-certified abortion support groups, and other reproductive health and rights groups and advocates who support people seeking abortions. The referral sources identified potential participants, informed them about the study, and shared a flyer with basic information about the study, including contact information. Interested candidates could contact or consent to be contacted by the research team, who screened them for eligibility and scheduled a telephone or in-person interview, according to their preferences and their and the researchers' availability. The interviewer, experienced in qualitative research of sensitive subjects, described the study, consented the participants, and answered any questions. Participants received a retail gift card valued at approximately U.S.$25 (20,000 Chilean pesos) for their participation. All study procedures were approved by the Allendale Institutional Review Board.

Interviews included open-ended questions about the participants' decisions to seek abortions, interactions with health care providers, and barriers to accessing abortion care, as well as the reasons for the denial and additional factors that framed their experiences. Interviews lasted ∼1 h and were conducted in Spanish, audio-recorded, and transcribed.

While we aimed to interview up to 15 participants, we only recruited 4 people despite diligent and lengthy recruitment efforts. To accommodate our small sample size, we adopted a multiple-case study methodological and analytical framework and utilized a “replication” design to identify (dis)similarities among these four cases.^10^ The analytical team included all co-authors, all fluent in Spanish, current or former residents of Latin America, with expertise in reproductive health and abortion access in Latin America.^11^ All co-authors read, summarized, and discussed the interview transcripts, developed a code list, and coded the transcripts in Dedoose.^12^ We conducted all data analysis in Spanish to avoid meaning loss during translation.

The first author (D.S.-B.) conducted the final formal synthesis of findings. We performed an inductive analysis, informed by grounded theory approach and time-series analyses in chronological sequences, in which abortion access was traced from pregnancy diagnosis to resolution of the pregnancy. Categories and themes emerged through inductive analysis and comparing codes within and between cases.^13,14^ Coding was conducted first from the transcripts, then by comparing the first and second authors' coding, and finally by identifying higher levels of abstraction after all authors reviewed the preliminary themes and categories. After analysis, we translated the interview excerpts and general themes to English using two forward and back translations. We present quotes and excerpts to illustrate the themes, removing identifying information to protect the participants' confidentiality and privacy.

## Results

After 12 months of recruitment, we interviewed four eligible women with an average age of 28 years. Two lived in rural areas and two in urban settings (Table 1). Two participants described the index pregnancy as planned pregnancy, although all participants described the index pregnancy as wanted.

**Table 1.** Cases description

Case	Age	Education	OB history	Marital status	Region	DX	Insurance	Ground for legal abortion	Pregnancy outcome
Case 01	28	University	G2T1A1L1	Partnered	Santiago	12–13 weeks Hydrops fetalis	ISAPRE	Lethal fetal anomaly	Forced pregnancy until “legal” abortion under false DX of missed miscarriage during second trimester
Case 02	25	University	G1T0P1L1	Partnered until pregnancy notification	Santiago	Mother's chronic illness	ISAPRE	Maternal health indication	Forced pregnancy until week 29 due to emergency c-section because maternal life-threatening condition
Case 03	29	University	G1T0	Partnered	Concepción	13: Multiple fetal malformations due to trisomy 13	ISAPRE	Lethal fetal anomaly	Forced pregnancy until legal abortion during second trimester
Case 04	32	University	G3T1M2L1	Partnered	Santiago	11 weeks Hydrops fetalis	ISAPRE	Lethal fetal anomaly	Forced pregnancy until week 20 due to emergency c-section because intrauterine fetal death

Obstetric history abbreviations: Gravida (total number of pregnancies), Term births, Preterm births, Abortions, Miscarriage, Living children.

Instituciones de Salud Previsional (ISAPRE short form for social security health institutions) are a private health insurance system, currently composed of nine insurers.

DX, diagnostic.

### Case descriptions

#### Case 1

This participant's pregnancy was planned and desired. She first learned something was wrong at her 13-week ultrasound when the physician informed her that the fetus might have hydrops fetalis, a lethal fetal anomaly. The physician would not provide a formal diagnosis and instead referred her for another ultrasound and OB/GYN to review the ultrasound. That same day, she visited a third physician who confirmed that the fetus had hydrops fetalis and could not survive. Although she wanted an abortion, this third physician advised her that she was ineligible for a legal abortion because the pregnancy was not “abnormal” although the pregnancy was risky. A fourth physician confirmed that she was eligible for a legal abortion and referred her to a public institution where she finally received an abortion, although they gave her the false diagnosis of a missed miscarriage to avoid the cumbersome legal process.

#### Case 2

This 25-year-old participant was aware of the risk of pregnancy due to her chronic health condition and became pregnant unexpectedly. Initially, she planned to keep the pregnancy and closely monitor her symptoms until her doctor informed her that the pregnancy placed her life at risk. When she asked if she was eligible for a legal abortion, the physician did not recommend an abortion for moral reasons, changed his diagnosis, and now told her the pregnancy was going well. She consulted five OB/GYNs and her chronic illness specialist during the second trimester, all of whom said her pregnancy was going well and any threats to her life would occur during the third trimester. She submitted a formal request for a legal abortion to the ethics committee of a University hospital and she was informed that they do not provide abortions. Next, she requested and was denied abortion access at a private hospital, because she was unmarried and needed to take the responsibility for her immoral behavior.

The participant was ultimately hospitalized in a Catholic hospital due to complications and forced to continue her pregnancy until her health condition provoked a multisystemic failure at 29 weeks' gestation, where she was stabilized and delivered a preterm birth by cesarean section.

#### Case 3

This participant was a 29-year-old woman living in southern Chile with her long-term partner. The pregnancy was unplanned but desired. Following her first ultrasound, her OB/GYN referred her to another OB/GYN for another ultrasound earlier than the usual 13 weeks, who identified severe lethal congenital malformations, including structural abnormalities to the heart. While she was eligible for a legal abortion at this moment, the physician recommended her to wait and get another ultrasound when the heart was fully developed. He also ordered a chorionic villus biopsy to confirm the diagnosis, which was not covered by her insurance.

After waiting 11 days, they told her to wait 60 additional days to confirm the diagnosis. Feeling distraught by the delay, she found a female OB/GYN who supported her and pressured the laboratory to release the biopsy results, which were eventually sent to the previous physician but not the new OB/GYN. Upon obtaining a trisomy 13 diagnosis and confirmation that the fetus could not survive, she decided to end the pregnancy. Days before the abortion, her new OB/GYN told her to obtain the medications on her own to end the pregnancy because the hospital was out-of-stock. With assistance from friends and a feminist organization, she obtained the medications and the Ob/GYN was able to provide the abortion.

#### Case 4

A 32-year-old woman living in the capital with her partner was trying to get pregnant for ∼1 year, had a history of miscarriage, and was receiving care to prevent a subsequent miscarriage. At her 11-week ultrasound, the fetus was diagnosed with hydrops fetalis and two cranial tumors with no chance of survival, later confirmed by genetic testing. She asked her physician about obtaining a legal abortion, and the physician submitted the case to the ethics committee at a public hospital. After waiting several weeks for a response, the woman decided to seek abortion care at a private hospital, where they denied her request because the fetus's heart was still developing.

Further attempts to obtain abortion at three different hospitals were also denied. In addition, she consulted a fetal cardiologist, who would not formally diagnose the lethal malformation while the heart was still developing. Ultimately, after several more weeks, the initial hospital's ethics committee denied her request and concluded that her pregnancy was “going well” despite her diagnosis. She was forced to continue the pregnancy until 20 weeks' gestation, when she had an emergency cesarean section following intrauterine fetal death.

### Description of themes

Five themes emerged during the analysis: (i) disparate levels of social support in accessing abortion, (ii) abundant access barriers, (iii) forced pregnancy, (iv) abortion stigma, and (v) a failure of the law to provide access to abortion.

#### Disparate levels of social support in accessing abortion

All the participants desired abortion to exercise their reproductive and bodily autonomy. Failed attempts to seek abortion care that was protected under the law forced them to find alternative settings and providers willing to provide them abortion care, which pushed one to obtain care outside of Chile's legal structure, one to obtain care under a false diagnosis, and two to risk their lives by carrying the pregnancy to term.

Participants considered, with support from friends and relatives, traveling internationally to get an abortion. For some of them, pursuing an abortion involved the whole family; for others, this same search threatened a loss of kinship because of the different ideologies around abortion. The participants' loved ones voiced a range of opinions, from support to opposition, even when abortion was a means to avoid future suffering of the fetus. The lack of familial support led some participants to external support networks and health care providers who made them feel recognized and offered them answers and hope.

Two participants accessed safe abortions, although beyond the terms of the law. The other two felt forced to continue their pregnancies, which ended in a stillbirth and premature birth (with additional complications), respectively.

“The thing is that I had a cesarean section, and I had asked for a cesarean section so that I could say goodbye. […] [It was] the 20^th^, almost the 21^st^ week. And then I asked them to pass it to me. And the minute they took [the baby] out, they left her on a tray next to me […] and she burst. […] They made her suffer unnecessarily. […] They said, ‘her lungs are full of fluid, her kidney is full of fluid, her heart is full of fluid,’ and you know that she is bursting inside you.” (Case 4)

#### Abundant access barriers

The legal process did not facilitate safe and efficient access to care for any of the participants, but rather imposed barriers and delays as institutions and providers evaded the law. Two participants reported feeling frustrated because it seemed impossible to obtain a legal abortion due to many bureaucratic obstacles, including a limited number of facilities equipped to provide the formal diagnoses required to initiate the legal abortion process, the need to get the diagnosis confirmed by a medical team and the abortion approved by institutional ethics committees, and few providers willing to provide care. The cumbersome legal process and legal loopholes resulted in providers and institutions denying people the care to which they were legally entitled.

Participants described how people with more resources could bypass many of these barriers by accessing care directly with private providers outside of the legal structure.

“I think that the law was made so that people who have a different economic status go to the [name of private clinic], have their procedure [outside the legal structure], and bye-bye. So, what, it takes you two days for the [diagnostic] result you need, maybe they even have it earlier because you had it done there—I don't know, who knows—and the doctor will take you to the operating room. I mean, that's why they made the law. I felt a little bit that in the public system, you have to wait for your turn after a long wait, and in the private system, you have to pay for it.” (Case 1)

Moreover, the participants described feeling that providers were insensitive to their situations. The physicians they encountered made them feel ignored, judged, and stigmatized. They further described the risk of being reported to the authorities when seeking abortion outside the law.

“As they say here in Chile, I needed to [deal with it] for reasons of morals—yes, morals. That's what that physician said…He said he was not going to suggest going for a legal termination of pregnancy, even if I had legal grounds, that because of his morals, he was not going to do it.” (Case 2)

#### Forced pregnancy

All four participants felt forced to remain pregnant and described how health care providers preferred to monitor their respective situations, whether waiting for fetal demise or for their own health to worsen. One participant related how the physicians would only perform an abortion in the case of a life-threatening emergency, disregarding the pregnancy's deleterious and potentially permanent impact on her health. According to the participant with a chronic condition who sought an abortion because of the risk to her life:

“My obstetrician said, ‘There is no way out. Here, you have to go ahead, and I am going to form a team with your [chronic illness specialist], and we are both going to follow you.’ So I assumed that this is the path I must follow—I had no option—with this team of the [chronic illness specialist] and the obstetrician, who told me that if I reached a life-threatening situation, they would be able to interrupt the pregnancy. But as long as I do not reach this situation—for example, a cardiac arrest (a cardiac arrest!)—my [chronic illness] doesn't count. A cardiac arrest was the only way I could have access to a legal termination of pregnancy.”(Case 2)

Altogether, these women reveal how they felt controlled by the government, health care institutions, ethics committees, conscientious objection, and provider preferences and deprived of their reproductive and bodily autonomy. Despite the severity of her health condition one participant never found a health care institution or provider to give her the care she wanted. By contrast, the experience of being denied a legal abortion caused physical and emotional harm, (re)victimization, and trauma.

“The only thing they are telling me is that I have to wait for my baby to die, whatever week it is […] that's going to be the course. I told him I don't…I don't feel capable of living through a pregnancy like this.” (Case 1)

#### Abortion stigma

The participants described the emotional consequences of the trauma from being denied a legal abortion. They explained how difficult it was to cope with the grief of having lost a desired pregnancy or, even worse, that the baby suffered, and they were unable to prevent its pain. The participants resisted their suffering and tried to be brave, particularly when their family members were also grieving the loss.

Some participants mentioned feeling responsible for not preventing fetal anomaly or guilty due to the judgment of health care professionals and family. This guilt and judgment contributed to the feelings of solitude. Furthermore, they reported constant fear of not being able to have an abortion, which ultimately became a reality for two participants. Ideas and perceptions of dehumanization and objectification were also recurrent, manifesting as a sense of complete loss of autonomy and agency to others who reduced their value to that of an incubator.

“I really felt that many times I was told that ‘you don't matter, you are not important, what you think, what you feel, no—the only thing you are responsible for here is taking that life forward.’ In the end, you don't matter; it only matters giving birth beyond consequences.” (Case 2)

Under the law, anyone seeking an abortion has the right to receive social, emotional, and informational support.^15^ Yet the participants shared that this support was barely available or of poor quality.

“I asked for accompaniment [social and emotional support]. It never came. And I asked until the last day, the accompaniment never came. I don't think they made the request either.” (Case 4)

#### A failure of the law to provide access to abortion

All participants held the profound belief that the current abortion law is ineffective and nonfunctional. They felt abandoned without legal protection and that nothing had changed in practice since the law changed. The participants described how their abortion requests should have been protected under the legal grounds, although they all were denied abortions by the hospitals' ethics committees, which have the authority to grant an abortion. Two participants described their experiences with committee members judging their sexual behavior and using these judgments to inform their ruling, even when one of their lives was in danger. According to this participant, she was scolded by the committee: “if she is pregnant, it's because she wasn't taking care of herself [practicing abstinence or using contraception] and, well, she'll just have to deal with it.” (Case 2)

Moreover, the participants described how the physicians who evaluated their cases and the institutions where they accessed abortion care interpreted the law differently, as exemplified by one who was forced to continue her pregnancy until she finally obtained an abortion from a physician outside the legal structure.

“On the other hand, I am angry—angry for feeling that the State does not protect you, that it is a law that does not work […] I felt like I want to shout in their face that their law does not work, that I just experienced it a week ago and that their law does not work.” (Case 1)

### Discussion and health equity implications

This study deepens our understanding of the experiences of people who sought and were denied legal abortions in Chile, soon after decriminalization of abortion in three limited circumstances. We found deep-rooted and persistent abortion stigma in the participants' experiences, as well as hospital systems, and individual providers unwilling to comply with the new law, obstructing and denying eligible people legal health care. Similar experiences of abortion stigma were described by women accessing legal abortion care in Uruguay.^16^ The participants' narratives also illustrated the barriers that may redirect people to seek abortion care outside of the legal structure. Our study highlights that pregnant-capable people face innumerable forms of gender-based discrimination, disparities, and inequalities and that have severe life-threatening consequences.^17,18^

Participants described providers who obstructed and did not facilitate abortion care, although the motives were unclear. Other research suggests that providers from other countries in Latin America where abortion is heavily restricted avoid providing care due to fear of legal problems, confusion about the law, and lack of training in abortion care. The barriers to accessing abortion described by our study participants echo prior research in Chile where in-depth interviews with clinicians (medical doctors, nurse midwives, social workers, and psychologists) and MOH officials about the implementation of Chile's abortion law highlighted how lack of awareness about the law among institutions, providers, and the general public; lack of provider training, abortion stigma, and intersectional discrimination generated barriers impeding access to abortion.^19,20^ Clinicians in Chile may need extensive support through proven interventions to reduce stigma and increase training and understanding about the proper implementation of the law.^21^

We also found that women's sexual behavior was stigmatized, as seen by the one woman who was denied care because she had sex outside of marriage. Similarly, a 2015 study in Chile found that abortion is stigmatized because it is viewed in some societal spheres as a means for women to behave immorally by allowing them to end pregnancies that occur outside of marriage.^22^ In addition to a widespread rejection of abortion access due to the aforementioned factors, information scarcity and unskilled abortion providers also present access barriers to legal abortion for pregnant people.^23^

Abortion is still seen as a major crime across Latin America; these beliefs result from the region's conservative political heritage, religiosity, and social stigma toward those who request and those who provide abortions.^24^ Therefore, Chile's governmental and health care institutions must find effective ways to implement the abortion law and make this effort a priority. In a similar study conducted in Colombia, where abortion during the first 24 weeks of pregnancy is decriminalized, the authors recommended promoting social awareness and general education about abortion access, providing support and enabling access when abortion rights are denied, and training providers to focus on compassionate care. Implementing these strategies will ensure that pregnant-capable people have access to “timely, safe, effective, and non-judgmental abortion care when needed.”^25^

Promptly after the abortion ban was lifted in Chile, a conservative president was elected, whose term coincided with our research. While access to legal abortion remains poor today,^3,4^ the incumbent government, led by a new progressive president, has shown a commitment to increasing access to abortion^5^ and backed a constitutional referendum that, had it passed, would have enshrined the legal right to abortion.^23–25^

Within the ongoing discourse surrounding abortion access, we find a need to address the persistent weaknesses in the implementation of legal reform in Chile as stated by the participants in our study, the law was not effective. We found that the abortion law in Chile fell short and did not ensure that people had access to the health care they were entitled to due to abundant barriers, providers' unwillingness to provide abortion care or the formal diagnoses needed to confirm eligibility for abortion under the law, and social stigma related to abortion. The participants in our study shared experiences about how the providers did not adhere to their legal and ethical obligation of providing access to abortion according to the law, to provide formal diagnoses, and sometimes ethics is used as an excuse to avoid the provision of abortion. Participants' stories revealed a significant gap between legal mandates and their practical implementation.

The consequences of the failure of providers to adhere to the law had serious, sometimes life-threatening, implications on the health and well-being of the people legally entitled to abortion. The stories of these four women highlight the systemic deficiencies and provider negligence within the framework of abortion laws. Our findings have implications for other countries in the region that have recently reformed their laws to expand access to abortion. Future research will need to examine the implementation of legal reform in those countries closely.

There are limitations to this study. The sample size of this study is small, and no people seeking abortion due to rape or incest were interviewed. Low participation rates may be attributed to abortion denial being an uncommon experience or, more likely, to pervasive abortion stigma, fear of judgment, or fear of criminalization and of being reported to the authorities, inhibiting people from sharing their experiences. Nevertheless, these four cases show that stigma and fear run deep and the risk of discussing their experiences are potentially high in a society with deeply rooted gender inequalities. The four participants in this study likely exemplify the struggles of many more pregnant women in Chile. Further studies must continue to explore the experiences of pregnant people seeking access to legal abortion and include an assessment of the effectiveness of legal reform.

Our findings underscore how laws expanding access to abortion do not necessarily ensure access when abortion stigma is deeply embedded in the health care system. Our findings can be applied to other countries in the region, such as Argentina, Colombia, and Mexico, which have recently expanded access to abortion.

By contextualizing the Chilean experience within this broader international framework, we gain valuable insights that can guide policymakers, activists, and stakeholders seeking seamless implementation of legal reform across diverse environments. To prevent the failure of abortion laws and the systems established to support them, collaboration between advocacy groups, health care providers, and policymakers is imperative. Robust education campaigns can raise awareness about the legality and importance of abortion access, while professional training can equip health care providers with the necessary skills and empathy to offer safe, supportive, and person-centered care that includes not only physicians but also nurses, midwives, social workers, and psychologists.^26–28^

## Conclusion

The cases shown in this study illustrate the experiences that women may face when seeking access to legal abortion in Chile. Furthermore, the barriers and stigma described by the participants mirror the religious and sociopolitical landscape of the region. The inability of the Chilean government to uphold the recent legislation, coupled with providers not adhering to the stipulated regulations, underscores the necessity for these stakeholders, alongside civil society, to collectively prioritize awareness of and accessibility to abortion. This emphasis should extend beyond the restricted scope of current abortion provisions and encompass a more comprehensive approach that recognizes abortion as an integral component of reproductive health care, an indispensable element of bodily autonomy, a non-negotiable pillar of safeguarded human rights, and guarantees the right to reproductive choice for all Chilean residents.

## Abbreviations Used

DX: diagnostic
MOH: Ministry of Health
OB/GYN: obstetric gynecologist
